# Impact of Dietary Lavender Essential Oil on the Growth and Fatty Acid Profile of Breast Muscles, Antioxidant Activity, and Inflammatory Responses in Broiler Chickens

**DOI:** 10.3390/antiox11091798

**Published:** 2022-09-13

**Authors:** Shimaa A. Amer, Ahmed A. A. Abdel-Wareth, Ahmed Gouda, Gehan K. Saleh, Arwa H. Nassar, Wafaa R. I. A. Sherief, Sarah Albogami, Shimaa I. Shalaby, Aaser M. Abdelazim, Mosleh Mohammad Abomughaid

**Affiliations:** 1 Department of Nutrition & Clinical Nutrition, Faculty of Veterinary Medicine, Zagazig University, Zagazig 44511, Egypt; 2 Department of Animal and Poultry Production, Faculty of Agriculture, South Valley University, Qena 83523, Egypt; 3 Animal Production Department, Agricultural & Biological Research Division, National Research Center, Dokki, Cairo 11865, Egypt; 4 Biochemistry Department, Animal Health Research Institute (AHRI) (Mansoura Branch) Agriculture Research Center (ARC), P.O. Box 246, Dokki, Giza 12618, Egypt; 5 Food Hygiene Department, Animal Health Research Institute (AHRI) (Mansoura Branch) Agriculture Research Center (ARC), P.O. Box 246, Dokki, Giza 12618, Egypt; 6 Animal Wealth Development Department, Faculty of Veterinary Medicine, Zagazig University, Zagazig 44511, Egypt; 7 Department of Biotechnology, College of Science, Taif University, P.O. Box 11099, Taif 21944, Saudi Arabia; 8 Physiology Department, Veterinary Medicine Faculty, Zagazig University, Zagazig 44511, Egypt; 9 Department of Basic Medical Sciences, College of Applied Medical Sciences, University of Bisha, Bisha 61922, Saudi Arabia; 10 Department of Medical Laboratory Sciences, College of Applied Medical Sciences, University of Bisha, Bisha 61922, Saudi Arabia

**Keywords:** antioxidants, blood biochemistry, broilers, essential oil, meat quality

## Abstract

This study aimed to investigate the impact of dietary addition of lavender essential oil (*Lavandula angustifolia* L.) (LEO) on the growth performance, tissue histoarchitecture, and fatty acid profile in breast muscles, as well as blood biochemistry and immune expression of pro-inflammatory cytokines of broiler chickens. A total of 200 three-day-old broiler chickens (average body weight 101.3 ± 0.24 g) were assigned to a completely randomized design consisting of four dietary treatments (*n* = 50 per treatment, each replicate consisting of 10 birds) that included lavender essential oil at concentrations of 0 (control group), 200, 400, and 600 mg Kg^−1^ diet. The experiment lasted for 35 days. The results revealed that supplementation of lavender essential oil at 200, 400, or 600 mg/kg in broiler diets had no effect (*p* > 0.05) on the growth performance throughout the experimental periods (3–10, 11–23, and 24–35 days of age). According to the broken line regression model, the optimal level for dietary LEO addition was the 460 mg kg^−1^ diet based on the total body weight gain and feed conversion ratio results. The diets supplemented with lavender essential oil had no effect (*p* > 0.05) on the percentages of carcass yield or internal organs. Dietary addition of LEO significantly increased the percentages of omega-3 polyunsaturated fatty acids PUFA (n-3), omega-6 polyunsaturated fatty acids (n-6), and the n-3/n-6 ratio (*p* < 0.05) in the breast muscles of chickens in a level-dependent manner. The blood concentration of alanine aminotransferase was significantly increased in lavender essential oil at 600 mg kg^−1^ compared with other treatments. The dietary addition of LEO at 200, 400, and 600 mg kg^−1^ significantly reduced the malondialdehyde level. Still, they significantly increased the serum enzyme activities of total antioxidant capacity, catalase, superoxide dismutase, and the pro-inflammatory cytokine (interleukine-1 beta and interferon γ) compared with the unsupplemented group. The LEO-supplemented groups showed normal liver histomorphology as in the control group. However, the immunoexpression of the pro-inflammatory cytokine transforming growth factor β was significantly increased by increasing the level of LEO. It can be concluded that lavender essential oil can be included in broiler chicken diets up to 460 mg kg ^−1^ with no positive effect on the bird’s growth. It can improve the antioxidant capacity and enrich the breast muscles with PUFA. An increased level of supplementation (600 mg kg^−1^) increased the inflammatory responses in broiler chickens.

## 1. Introduction

Over the past ten years, poultry farmers have placed a high premium on bird welfare and food safety. Consumer pressure forces the abandonment of synthetic feed additives and the mitigation of excessive breeding practices to protect the environment. Differentiating herbs that affect fatty acid characteristics and antioxidant status is a goal of several researchers [1,2,3]. The quality of poultry meat is one of the main factors affecting consumer health [4,5]. The breast meat can be considered an important element of a healthy diet [6] because it contains more PUFA and less saturated fatty acids (SFAs) than meat from other types of animals, such as beef and lamb [7]. Moreover, its global consumption is increasing as it fits perfectly with modern cooking styles where easy and quick cuisine is desired.

The essential oil of herbs has various biological effects, including immune-modulating, antibacterial, and antioxidant characteristics [2,8,9,10]. Developing essential oil feed additives as a possible substitute for antibiotic growth promoters has recently gained more attention [5,11,12]. Numerous studies have also been conducted on how dietary essential oil affects poultry productivity, although the outcomes have been varied [13,14,15]. However, the response of poultry to the essential oil differed depending on many variables, including chemical composition, administrative levels, durations, techniques, and the age of the birds [16,17,18]. Thus, the essential oils’ activities rely on their composition, functional groups, and synergistic interactions of the components.

Scientific indications, including essential oils of sage, rosemary, and oregano, have proven that they can reduce oxidative damage to lipids in serum and edible tissue while improving poultry meat’s physical and sensory characteristics, especially broiler chicken [19,20]. Despite extensive studies and attempts to comprehensively characterize phenolic compounds obtained from Labiate family plants, understanding the mode of action and applications using lavender is still undeveloped.

One alternative to antibiotics that can be added to feed is lavender (*Lavandula angustifolia* L.). It is a well-known medicinal and aromatic herb member of the “Labiatae” family. Antioxidant, antifungal, antibacterial, antiviral, spasmolytic, and sedative effects are present in this herb [21,22]. Lavender essential oil, one of the most useful aromatherapy oils, has antibacterial and antifungal activities, mainly owing to the principal components such as lavandulol, linalool, linalyl acetate, eucalyptol, or geraniol [23]. Furthermore, it contains high levels of polyphenols such as flavonoids, which have a wide range of biological and chemical activities with radical scavenging properties [24]. It is established that the chemical composition of LEO depends on the environment, genotype, extraction, and processing methods [25]. Adding lavender essential oil to the water for broiler chickens enhanced the total antioxidant status [26]. In vitro studies have studied the anti-inflammatory [27], antioxidant [28,29], antimicrobial, and antifungal properties of lavender essential oil [27,28]. It has been reported that adding lavender essential oil to the drinking water of broiler chickens can increase the growth rate and enhance gut microbiota balance by lowering colonization of pathogenic bacteria on the side of useful bacteria [30].

Nevertheless, little research is currently available on the impact of lavender essential oil on broiler production efficiency and meat quality. Accordingly, this study aimed to assess the effectiveness of lavender essential oil on the growth performance, tissue histoarchitecture, fatty acid profile of the breast muscles, blood biochemistry, antioxidant activity, and immune expression of pro-inflammatory cytokines in broiler chickens.

## 2. Materials and Methods

### 2.1. Gas Chromatography-Mass Spectrometry (GC-MS) Analysis of Lavender Essential Oil

The chemical composition of LEO was performed using a Trace GC1310-ISQ mass spectrometer (Thermo Scientific, Austin, TX, USA) with a direct capillary column TG–5MS (30 m × 0.25 mm × 0.25 µm film thickness). The column oven temperature was initially held at 50 °C and then increased at 5 °C/min to 230 °C for 2 min, increased to the final temperature of 290 °C at 30 °C/min, and then held for 2 min. The injector and MS transfer line temperatures were kept at 250 and 260 °C, respectively; helium was used as a carrier gas at a constant flow rate of 1 mL/min. The solvent delay was 3 min, and diluted samples of 1 µL were injected automatically using Autosampler AS1300 coupled with GC in the split mode. In full scan mode, EI mass spectra were collected at 70 eV ionization voltages over m/z 40–1000. The ion source temperature was set at 200 °C. The components were identified by comparison of their retention times and mass spectra with those of WILEY 09 and NIST 11 mass spectral databases.

### 2.2. Birds, Experimental Design, and Diets

This research was conducted in a poultry research unit at the faculty of veterinary medicine, Zagazig University, Egypt. All experiment procedures were approved by the ZU-IACUC committee (approval no. ZU-IACUC/2022).

Two-hundred one-day-old Ross 308 broiler chicks were purchased from a commercial hatchery (Dakahlia Poultry, Mansoura, Egypt). Before the experiment, birds underwent a 3-day adaption period to gain an average body weight of 101.3 ± 0.24 g. Then, they were divided into four experimental groups at random, each with five replicates (10 chicks/replica). For 35 days, birds were fed basic diets supplemented with LEO (lavender essential oil, ORGANIC EGYPT, Cairo, Egypt) at four levels: 0, 200, 400, and 600 mg Kg^−^^1^. The proximate chemical composition of the basal diet is shown in Table 1. The managerial conditions and the experimental diets were carried out following Ross 308 broiler nutrition specifications AVIAGEN [31].

### 2.3. Growth Performance

The initial body weight (BW) was determined at the beginning of the experiment. Body weight and feed consumption were assessed at the end of each feeding period to determine BW gain (BWG), feed intake, and feed conversion ratio (FCR). The number of birds that died throughout the trial period was divided by the number of birds used to compute the number of dead birds. To account for bird mortalities, BW and feed consumption were adjusted.

The feed conversion ratio $\mathrm{FCR}=\frac{\mathrm{FI}(g)}{\mathrm{BWG}(g)}$

The relative growth rate (RGR) was calculated using the equation described by Brody [32].

$$RGR=\frac{W2-W1}{0.5\;\left(W1+W2\right)}\times 100\;$$

W1 = the initial live weight (g) and W2 = the live weight at the end of the period (g).

The protein efficiency ratio (PER) was determined according to McDonald et al. [33].

$$PER=\frac{Live\;weight\;gain\;\left(g\right)\;}{protein\;intake\;\left(g\right)}\;$$

### 2.4. Chemical Analysis and Fatty Acid Composition of the Breast Muscle

Samples of the feed and breast muscles were examined for moisture using oven drying (method number 930.15), ash using incineration (method number 942.05), protein using Kjeldahl (method number 984.13), and EE using Soxhlet fat analysis (method number 954.02). Breast muscle samples (five samples/group) were collected at the end of the trial. Chloroform/methanol (2:1, *v*/*v*) was used as the solvent to extract the oils from the breast muscle [34]. According to AOAC [35], the fatty acids in the extracted oil and the chemical composition of the breast muscle (dry matter, fat, crude protein, and ash content percent) were determined.

### 2.5. Carcass Characteristics

Following the recommendations of the American Veterinary Medical Association, nine chicks in each group were terminated at the end of this study using cervical dislocation [36]. The weight of the hot carcass was determined. The plucked and eviscerated carcasses were stripped of their feet; feathers; heads; and internal organs such as the liver, heart, gizzard, spleen, digestive system, and abdominal fat before being weighed. The weight of the carcass was measured, and the percentage of dressing was calculated as follows:

$$Dressing\;\%=\frac{\mathrm{Carcass}\;weight\;\left(g\right)\;}{\mathrm{Live}\;\mathrm{BW}\;\left(g\right)}\;\times \;100\;$$

### 2.6. Blood Biochemical Assay

At the end of the experimental period, blood samples (*n* = 10/group) were collected from the carotid artery after birds were euthanized using cervical dislocation, according to the American Veterinary Medical Association guidelines [36]. Serum was obtained by centrifugation at 3000 rpm for 10 min, and the generated serum was stored at −20° until being used in the biochemical assays.

Using colorimetric diagnostic kits (Biodiagnostic Co., Giza, Egypt), liver enzymes such as aspartate transaminase (AST) and alanine transaminase (ALT) and kidney function such as creatinine and urea were assessed following the manufacturer’s instructions.

### 2.7. Inflammatory and Antioxidant Indices

Specific ELISA assay kits (MyBioSource, San Diego, CA, USA) were used to quantify interleukin 1β (IL1β) and INF-γ (Cat. No. MBS2024496 and MBS700243, respectively). Malondialdehyde (MDA), total antioxidant capacity (TAC) levels, the activities of catalase (CAT), and superoxide dismutase (SOD) were estimated following the techniques of Mcdonald and Hultin [37], Rice-Evans and Miller [38], Aebi [39], and Nishikimi et al. [40], respectively, using MyBioSource ELISA Kits (Cat. Nos. MBS2700234, MBS038818, and MBS705758, respectively).

### 2.8. Histological and Immunohistochemical Examination

Samples (three samples/group) were taken from the chicken’s liver and were fixated in neutral buffered formalin (10%) for analysis. Briefly, specimens were subjected to ascending grades of ethanol (75–100%) for dehydration. These specimens were placed in xylol I and II, embedded in paraffin, and then sliced using a microtome (Leica RM 2155, England) into 4 µm cross-sections and longitudinal sections. They were stained using hematoxylin and eosin (H&E) [41]. An AmScope 5.0 MP microscope digital camera selected on high power field (100× and 400× magnification) was used to take images of each animal in each group (25 images for each group). Stained sections were examined for circulatory disturbances, inflammation, degenerations, apoptosis, necrosis, and other pathological changes in the examined tissues.

The inflammatory responses of broiler chickens following feeding on LEO were explored in the leucocytic populations of the liver and spleen using transforming growth factor-β (TGF-β). At the end of the experiment, liver and spleen samples (three samples/group) were collected to examine the immunoexpression of TGF-β, according to Saber et al. [42]. An endogenous peroxidase blocking reagent containing hydrogen peroxide and sodium azide (DAKO peroxidase blocking reagent, Cat. No. S 2001) was used to incubate tissue sections. Then, one to two drops of the supersensitive primary monoclonal antibody against TGF-β (Cat. BAF240, Novus Biologicals, Briarwood Avenue, Centennial, CO, USA) were added to these sections; then, the slides were counterstained using hematoxylin and visualized under the microscope. Morphometric analysis using Image J software (ImageJ bundled with 64-bit Java 1.8.0_172, National Institutes of Health, Maryland, United States) was adopted to accurately estimate different immune-positive cells and their percentages per three high power fields (HPFs) in the spleen and liver of different experimental groups [43].

### 2.9. Statistical Analysis

The statistical analysis was performed using a completely randomized design and the general linear model (GLM) procedure of SAS 9.2. Pens were used as the experimental units throughout the entire investigation. The linear and quadratic effects of increasing inclusion levels were determined using orthogonal polynomial contrasts. Tukey’s test compared the differences between the means at a 5% probability. Variation in the data was expressed as pooled SEM, and the significance level was set at *p* < 0.05. The broken line regression with Tukey’s test considered information on BWG and FCR for determining the optimum supplementation level of LEO.

## 3. Results

### 3.1. Chemical Composition of Lavender Essential Oil

The bioactive compounds identified in the LEO by the GC-MS technique are shown in Table 2. The main bioactive compounds identified were α-pinene, acetic acid linalool ester, terpineol, α-linalool, eucalyptol, phellandral, geranyl vinyl ether, nerolidyl acetate, and acetic acid. The fatty acids identified in LEO were 9,12,15-octadecatrienoic acid (α-linolenic acid), arachidonic acid, 3,7,11-tridecatrienoic acid, 5,10-undecadienoic acid, z-8-methyl-9-tetradecenoic acid, trans-2-undecenoic acid, 13-docosenoic acid, and 11-octadecenoic acid.

### 3.2. Growth Performance

**Figure 1:**
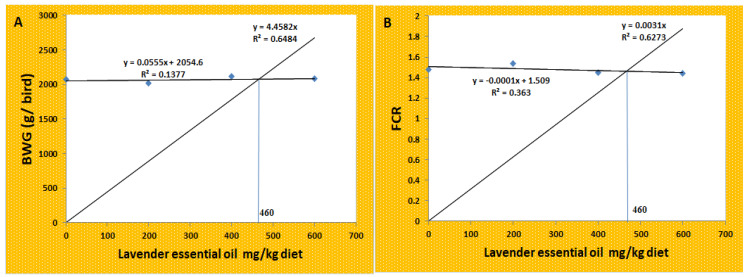
Broken line regression model shows the optimum level of dietary addition of LEO according to the total (**a**) BWG and (**b**) FCR.

Table 3 overviews lavender essential oil’s effects on broiler chickens’ growth performance. The absence of fatalities, diarrhea, and other symptoms in the treatment groups suggests that the therapies had no adverse effects on the broilers’ health. The findings showed that adding 200, 400, or 600 mg/kg of lavender essential oil to broiler diets had no effect (*p* > 0.05) on feed intake, BW, BWG, FCR, PER, and RGR when compared with the control across the course of the trial. Based on the BWG and FCR, the optimal level for dietary LEO addition was 460 mg kg^−1^ (Figure 1).

### 3.3. Carcass Characteristics

The effects of lavender essential oil at 200, 400, or 600 mg/kg on carcass and internal organs of broiler chickens at 35 days of age are presented in Table 4. The diets supplemented with lavender essential oil to broiler diets had no effect (*p* > 0.05) on the percentages of carcass yield or internal organs, including intestinal, liver, heart, gizzard, and spleen.

### 3.4. Chemical and Fatty Acid Composition of the Breast Muscle

Table 5 provides information on the breast muscle’s fatty acid and chemical composition. Compared with the control group, adding LEO had no appreciable impact on the amount of dry matter, fat, crude protein, and ash in breast muscles (*p* > 0.05). Dietary addition of LEO raised the percentages of alpha-linolenic acid (18:3 n − 3), eicosapentaenoic acid (20:5 n − 3), docosapentaenoic acid (22:5 n − 3), linoleic acid (18:2 n − 6), arachidonic acid (linear, *p* < 0.01), docosahexaenoic acid (22:6 n − 3) (linear *p* < 0.01, quadratic *p* = 0.02), total n-3 polyunsaturated fatty acids (linear *p* < 0.01, quadratic *p* = 0.03), total n-6 polyunsaturated fatty acids (linear *p* < 0.01), and the ratio of n − 3 to n − 6 (linear *p* < 0.01, quadratic *p* = 0.03).

### 3.5. Blood Biochemical Parameters

The blood-based biochemical parameters of broilers are presented in Table 6. The blood concentration of ALT was increased in lavender essential oil at 600 mg kg^−1^ level compared with the control (linear *p* = 0.03); however, AST, creatinine, and uric acid did not report significant changes compared with the control group.

### 3.6. Serum Antioxidant Activity and Inflammatory Responses

The serum antioxidant capacity and lipid peroxidation data from the broilers are shown in Table 7. The dietary supplementation with lavender essential oil at 200, 400, and 600 mg/kg significantly reduced the MDA (linear, *p* < 0.01) content, but significantly increased (linear, *p* < 0.01) the serum enzyme activities of TAC, CAT, SOD, IL1β, and IFN-γ compared with the unsupplemented group.

### 3.7. Histopathological Responses

**Figure 2:**
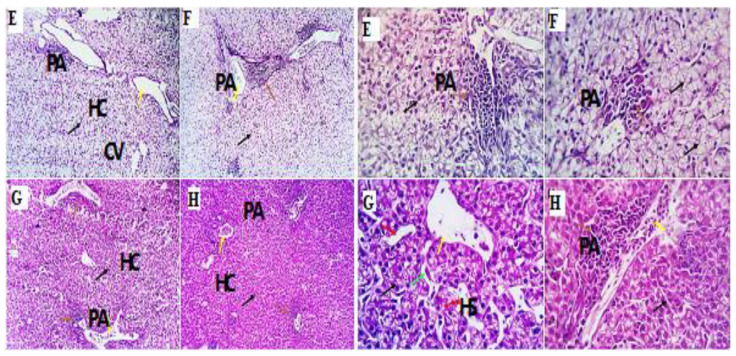
Photomicrograph from the liver of different experimental groups of chicken receiving 0 mg/kg lavender (**E**), 200 mg/kg (**F**), 400 mg/kg (**G**), and 600 mg/kg (**H**) showing the following: normal histological characterization of different structures, including portal area (PA, yellow arrow) hypertrophied Von Kupffer cells (green arrow), and hepatocytes (HC, black arrow), which are seen as small masses around the central veins (CVs), while a few round cells are seen as a natural immune response around the portal area (PA, yellow arrow). Mild dilation of hepatic sinusoids (HS, red arrow) is seen in the group (**G**). H&E ×100, 400.

Examined liver tissue from several experimental groups, treated with lavender extract at doses of 0, 200, 400, and 600 mg kg^−^^1^ for groups LEO0, LEO200, LEO400, and LEO600, respectively, revealed lobules made of hepatocyte groups. The interlobular branch of the hepatic artery, the interlobular branch of the portal vein, the bile ductulus, and less obvious lymphatic vessels and nerve branches were located near the periphery of each lobule. There was a huge central vein in the center of each lobule. Most of the hepatic parenchyma was made of rows of conical hepatocytes bordering the borders of elongate sinusoids. Hepatic plates were created by the hepatocytes joining together in a hexagonal pattern. The hepatic plates were ad hoc and positioned inward toward the central vein from the outer margin of each liver lobe. Very few lymphoplasmacytic aggregations (inflammatory cells) and mild portal vascular dilation were seen in the LEO600 group. Instead of being a harmful inflammatory process, aggregated portal inflammatory cells appear to be immune surveillance and protective mechanisms (Figure 2).

### 3.8. Immunohistochemical Analysis and Morphometric Measures

**Figure 3:**
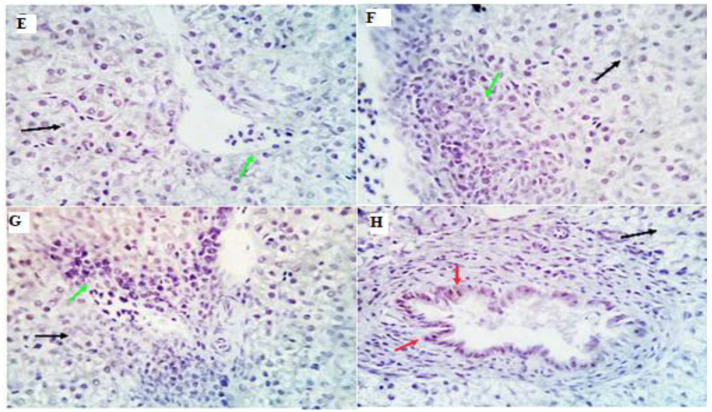
Immunostained positive TGF-β cells (red arrows) in the liver of different experimental chicken groups. (**E**): 0.08, (**F**): 0.45, (**G**): 0.93, and (**H**): 7.2% for LEO0, LEO200, LEO400, and LEO600 groups.

**Figure 4:**
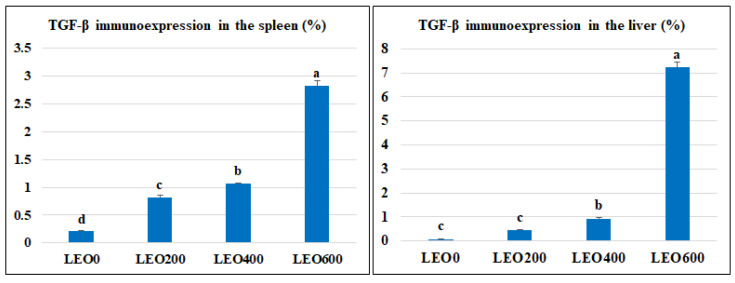
The morphometric analytic data in the spleen and liver of different experimental groups. LEO0, LEO200, LEO400, and LEO600: basal diets supplemented with 0, 200, 400, and 600 mg Kg^−^^1^ lavender essential oil, respectively. ^a, b, c, d^ Means carrying different superscripts are significantly different at (*p* < 0.05). TGF-β: transforming growth factor beta.

**Figure 5:**
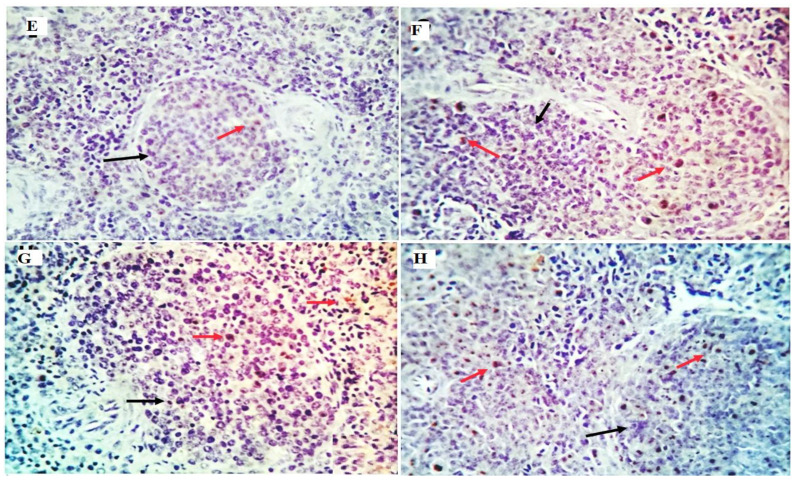
Immunostained positive TGF-β cells (red arrows) in the spleen of different experimental chicken groups (**E**): 0.21, (**F**): 0.82, (**G**): 1.06, and (**H**): 2.8% for LEO0, LEO200, LEO400, and LEO600 groups, respectively.

Examined sections from the liver of different experimental groups with morphometric analysis revealed an average percentage of positive cells per three high power fields (HPFs) to the pro-inflammatory marker (TGF-β) in different experimental groups as follows: 0.08, 0.45, 0.93, and 7.2% for LEO0, LEO200, LEO400, and LEO600 groups, respectively (Figure 3 and Figure 4). Meanwhile, examined sections from the spleen of different experimental groups with morphometric analysis revealed an average percentage of positive cells per three high power fields (HPFs) to the pro-inflammatory marker (TGF-β) in other experimental groups as follows: 0.21, 0.82, 1.06, and 2.8% for LEO0, LEO200, LEO400, and LEO600 groups, respectively (Figure 4 and Figure 5).

## 4. Discussion

Lavender essential oil is a mix of volatile organic compounds. In the present study, the main bioactive compounds identified in LEO by GC-MS analysis were monoterpenes such as α-pinene (32.24%), acetic acid linalool ester (32.24%), and α-linalool (16.06%) and monoterpenoids such as terpineol (16.06%), eucalyptol (11.74), and phellandral (10.91%). These active ingredients act as a digestion enhancer, balance the microbial ecosystem in the gut, and stimulate the secretion of endogenous digestive enzymes, thus increasing growth performance in poultry [44]. Previous studies demonstrated the antimicrobial activity of α-pinene, the main component of LEO (32.24%) [45,46]. Linalool has been shown to have appetizing properties and stimulate animal digestive processes [47]. However, our results indicated that broiler chickens fed diets supplemented with lavender essential oil exhibited no significant difference in growth performance during the experimental periods compared with broiler chickens fed unsupplemented diets. No mortalities were recorded among the experimental groups. Similarly, Küçükyilmaz et al. [48] reported no significant effect of LEO (24 and 48 mg/kg of diet) on broiler chickens’ FI, FCR, and mortality. Salajegheh et al. [49] showed that lavender powder (at a 1% level) improved the BWG and FCR during the grower, finisher, and whole rearing periods. Nasiri-Moghaddam et al. [50] reported increased BWG and reduced FCR of broiler chickens by dietary addition of LEO (350 mg/kg diet) at an age of 22 to 42 d.

Concerning carcass criteria in the current study, lavender essential oil addition to broiler diets did not affect the percentages of carcass yield or internal organs, including intestinal, liver, heart, gizzard, and spleen. This result was in agreement with the result reported by Salajegheh et al. [49], which showed no effect of lavender powder at 1% supplementation on the relative weight of broilers’ thighs, breasts, and internal organs at 42 days of age. Moreover, Nasiri-Moghaddam et al. [50] reported no effect of LEO on the relative weight of the gastrointestinal tract of broiler chickens.

The fatty acid profile of chicken meat is influenced by the diet of the birds [4] and genetic factors [51]. Chicken meat is richer in PUFA than other meat because the diet of broilers is usually rich in PUFA [52]. Lately, dietary actions to alter the fatty acid profile of meat have been a popular research topic [2,5,53,54]. Currently, the ways of enriching diets for birds with plant extracts have received much attention owing to the many beneficial uses of these extracts and, at the same time, the potential to increase production capacity and enhance poultry health [55]. In the current study, dietary LEO addition enriched the breast muscle with n-3 PUFA, particularly α-linolenic acid, eicosapentaenoic acid, docosapentaenoic acid, and docosahexaenoic acid, and n-6 PUFA, mainly linoleic acid and arachidonic acid. Its addition also increased the n-3/n-6 ratio in a dose-dependent manner, which has the advantage of increasing consumer acceptance. The breast muscle enrichment with PUFA in the LEO-fed birds can be attributed to the fatty acid composition of LEO represented by 9,12,15-octadecatrienoic acid (α-linolenic acid), arachidonic acid, 3,7,11-tridecatrienoic acid, 5,10-undecadienoic acid, z-8-methyl-9-tetradecenoic acid, trans-2-undecenoic acid, 13-docosenoic acid, and 11-octadecenoic acid. Kartikasari et al. [56] showed that enriching broiler chicken diets with short-chain n-3 PUFA (α-linolenic acid) lead to an increase in the percentages of long-chain n-3 PUFA (eicosapentaenoic acid (20:5 n − 3), docosapentaenoic acid, and docosahexaenoic acid) in the chicken tissues. However, the chemical composition of the breast muscles (dry matter, crude protein, fat, and ash content) did not differ from the unsupplemented group. Unfortunately, there are scarce studies on the effect of LEO on the fatty acid profile of breast meat. Hristakieva et al. [57] reported no significant effect of LEO on the fatty acid profile of breast muscles of broiler turkey.

The current findings showed that ALT was increased in lavender essential oil compared with the control in a level-dependent manner; however, AST and uric acid did not report any changes among treatments, but the values are within the normal physiological range [26,58,59]. These results are confirmed by the histomorphological examination of the liver, which appeared normal among the experimental groups. However, very few lymphoplasmacytic aggregations (inflammatory cells) and mild portal vascular dilation were seen in the LEO600 group. This inflammatory action can explain the increased ALT level in the serum by increasing the level of LEO.

As a crucial immune response of the organism, an extreme inflammatory response can trigger a significant increase in the cytokines, causing disturbance of the immune system and eventually permanent damage to the host [60]. Nitric oxide synthase, peroxidase oxygenase, and peroxidase activity can be increased by inflammatory processes, which can also cause the release of pro-inflammatory cytokines [61,62]. Essential oils can also influence the expression of pro-inflammatory genes, regulatory transcription factors, and signaling cytokines. The method may involve inhibiting the production and release of pro-inflammatory cytokines and inflammatory mediators, acting as an anti-inflammatory drug [63]. The present study showed increased inflammatory responses as a consequence of increasing the level of LEO feeding indicated by increased serum level of pro-inflammatory cytokines (IL1β and IFN-γ) and mild up-regulation of the immune expression of *TGF-β* in the liver and spleen. This suggests the immunostimulant effect of LEO on the innate immunity of birds through increasing the production of pro-inflammatory cytokines, which can stimulate inflammatory responses in chickens. These results confirm the histopathological examination of the liver, where we can find very few lymphoplasmacytic aggregations (inflammatory cells) and mild portal vascular dilation at the highest levels of LEO (600 mg Kg^−1^). Instead of being a harmful inflammatory process, aggregated portal inflammatory cells appear to be immune surveillance and protective mechanisms. The immunostimulant effect of LEO may be due to its bioactive compounds from monoterpenes and monoterpenoids [45,46,60].

Numerous extracts and essential oils of particular herbs contain antioxidant characteristics that have been demonstrated to be beneficial in delaying lipid peroxidation in oils and fatty diets. These properties have attracted the attention of many researchers [1,3,64,65]. In the current investigation, supplementing the diet with lavender essential oil at doses of 200, 400, and 600 mg/kg linearly decreased the MDA content while linearly increasing the serum enzyme activities of TAC, CAT, and SOD in comparison with the control group. These findings suggested that the phenolic compounds had a significant role in the essential oils’ ability as antioxidants. Lavender essential oil supplementation boosted the activities of SOD and GSH-Px and lowered the level of MDA in the serum [66]. Supplementing essential oils to broiler diets is a simple and convenient strategy to incorporate natural antioxidants into the meat. Essential oils could increase the oxidative stability of chicken tissues [66]. The antioxidant activity of LEO can be attributed to its content from monoterpenes such as α-pinene, acetic acid linalool ester, and α-linalool and monoterpenoids such as terpineol, eucalyptol, and phellandral, which were proven for their antimicrobial and antioxidant activity [67,68,69,70].

## 5. Conclusions

Lavender essential oil can be included in broiler chicken diets up to 460 mg kg ^−^^1^ with no positive effect on the bird’s growth. It can improve the antioxidant capacity without side effects on the carcass or internal organs. Feeding broiler chickens on diets containing LEO enriched the breast meat with polyunsaturated fatty acids, increasing consumer acceptance. Increased levels of supplementation (600 mg kg^−^^1^) increased the inflammatory responses in broiler chickens, indicated by higher serum levels of IL1β and IFN-γ and up-regulation of the immune expression of *TGF-β* in the liver and spleen.

## Figures and Tables

**Table 1:** The proximate chemical composition of the basal diet on a fed basis.

Ingredients	Unit	Starter (3–10 d)	Grower (11–23 d)	Finisher (24–35 d)
Corn 7.5% cp	%	56	59.3	62
Corn gluten meal 60% cp	%	3.92	5.27	6.07
Soybean meal 47% cp	%	33.33	28.1	23.82
Oil (soya)-e76	%	2.2	3	4
Calcium carbonate	%	1.2	1.2	1.1
Dicalcium phosphate 18%	%	1.5	1.4	1.3
DL-methionine 99%	%	0.4	0.3	0.33
Sodium bicarbonate	%	0.25	0.25	0.25
Broiler premix *	%	0.3	0.3	0.3
Salt	%	0.15	0.11	0.15
L-LYSINE HCL 98%	%	0.46	0.45	0.4
Antimycotoxin	%	0.1	0.1	0.1
L-THREONINE 98.5%	%	0.1	0.1	0.1
Choline 60	%	0.07	0.07	0.07
Phytase	%	0.005	0.005	0.005
Chemical analysis				
Moisture	%	11.25	11.26	11.19
Crude protein	%	23.01	21.52	20.15
ME (kcal/kg)	Kcal/kg	3003.88	3100.40	3200.84
Methionine	g/kg	7.20	6.10	6.26
Lysine	g/kg	14.65	13.14	11.63
Calcium	g/kg	9.40	9.04	8.32
Av. Phosphorus	g/kg	4.81	4.48	4.17

* Premix per kilogramme of diet: 1500 IU vitamin A; 200 IU vitamin D; 10 mg vitamin E; 0.5 mg vitamin K; 1.8 mg thiamine; 3.6 mg riboflavin; 10 mg pantothenic acid; 0.55 mg folic acid; 3.5 mg pyridoxine; 35 mg niacin; 0.01 mg cobalamin; 0.15 mg biotin; 80 mg iron; 8 mg copper; 60 mg manganese; 40 mg zinc; 40 mg; I, 0.35 mg; Se, 0.15 mg.

**Table 2:** The main compounds identified in LEO by GC-MS analysis.

Bioactive Compounds	Retention Time (min)	Peak Area %
α-Pinene	11.10	32.24
Acetic acid linalool ester	11.10	32.24
Terpineol, cis-α-	7.37	16.06
cis-α-Terpineol	7.37	16.06
α-linalool	7.37	16.06
Eucalyptol	5.30	11.74
Phellandral	8.20	10.91
Geranyl vinyl ether	11.99	2.25
Nerolidyl acetate	11.99	2.25
3,7,11-Tridecatrienoic acid, methyl ester	14.92	2.49
9,12,15-Octadecatrienoic acid	5.18	2.43
Isoborneol	5.18	2.43
Acetic acid	7.90	2.69
Desulphosinigrin	7.90	2.69
2,6-Octadien-1-ol, 2,7-dimethyl	11.99	2.25
1,6-Octadien-3-ol, 3,7-dimethyl-, formate	11.99	2.25
Arachidonic acid methyl ester	14.39	0.36
5,10-Undecadienoic acid, 2-methylene-, methyl ester	14.92	2.49
Z-8-Methyl-9-tetradecenoic acid	15.27	0.64
trans-2-undecenoic acid	15.27	0.64
13-Docosenoic acid, methyl ester	27.82	0.70
11-Octadecenoic acid, methyl ester	27.82	0.70

**Table 3:** Impact of lavender essential oil addition on the growth of broiler chickens.

Parameters	Lavender Essential Oil, mg/kg				SEM	*p*-Value	
	0	200	400	600		Linear	Quadratic
IBW (g)	100.2	101.5	101.6	101.9	0.24	0.28	0.25
Starter period							
BW (g)	316	324	328	330	3.17	0.17	0.64
BWG (g)	216	223	227	228	3.01	0.21	0.69
FI (g)	263	259	264	264	2.21	0.73	0.77
FCR	1.21	1.16	1.16	1.15	0.01	0.08	0.30
Grower period							
BW (g)	1107	1146	1170	1189	13.62	0.15	0.66
BWG (g)	790	822	842	859	11.66	0.18	0.72
FI (g)	1135	1124	1142	1116	18.22	0.82	0.86
FCR	1.43	1.36	1.35	1.29	0.02	0.05	0.89
Finisher period							
BW (g)	2175	2120	2214	2182	19.80	0.53	0.78
BWG (g)	1067	974	1043	993	22.68	0.47	0.65
FI (g)	1681	1740	1648	1616	36.05	0.43	0.57
FCR	0.77	0.82	0.74	0.74	0.01	0.30	0.48
Overall performance							
BW (g)	2174	2120	2214	2182	19.80	0.53	0.78
BWG (g)	2074	2019	2112	2080	19.82	0.55	0.77
FI (g)	3080	3124	3055	2996	49.41	0.54	0.65
FCR	1.48	1.54	1.45	1.44	0.02	0.38	0.56
PER	3.22	3.09	3.30	3.34	0.06	0.33	0.52
RGR	182.36	181.73	182.41	182.15	0.16	0.97	0.57

IBW: initial body weight, BW: body weight, BWG: body weight gain, FI: feed intake, FCR: feed conversion ratio, PER: protein efficiency ratio, RGR: relative growth rate.

**Table 4:** Impact of lavender essential oil addition on the carcass traits (%) relative to live body weight.

Parameters	Lavender Essential Oil, mg/kg				SEM	*p*-Value	
	0	200	400	600		Linear	Quadratic
Carcass	57.32	59.63	56.92	58.92	0.63	0.72	0.90
Intestine	5.41	5.56	5.72	5.52	0.19	0.81	0.70
Spleen	0.11	0.12	0.09	0.08	0.01	0.14	0.72
Bursa	0.11	0.13	0.14	0.11	0.01	0.90	0.50
Gizzard	2.32	2.16	2.12	2.27	0.10	0.84	0.54

**Table 5:** Impact of lavender essential oil on the breast muscle’s chemical and fatty acid composition.

Parameters	Lavender Essential Oil, mg/kg				SEM	*p*-Value	
	0	200	400	600		Linear	Quadratic
Dry matter %	71.02	72.13	73.68	74.24	0.53	0.28	0.37
Crude protein%	66.27	66.25	66.1	65.7	1.40	0.55	0.58
Fat %	5.92	4.9	6.0	5.8	0.30	0.76	0.64
Ash %	4.2	4.6	4.26	4.7	0.38	0.46	0.73
ALA %	0.02 ^b^	0.06 ^a^	0.06 ^a^	0.07 ^a^	0.007	<0.01	0.05
EPA %	0.02 ^b^	0.04 ^a^	0.04 ^a^	0.05 ^a^	0.004	<0.01	0.06
DPA %	0.01 ^b^	0.04 ^a^	0.05 ^a^	0.05 ^a^	0.005	<0.01	0.05
DHA %	0.01 ^b^	0.03 ^a^	0.03 ^a^	0.03 ^a^	0.003	<0.01	0.02
LA %	0.79 ^b^	0.83 ^ab^	0.85 ^ab^	0.88 ^a^	0.01	<0.01	0.81
AA %	1.19 ^b^	1.23 ^ab^	1.26 ^ab^	1.29 ^a^	0.01	<0.01	0.75
n-3 (%)	0.06 ^b^	0.18 ^a^	0.19 ^a^	0.21 ^a^	0.01	<0.01	0.03
n-6 (%)	1.98 ^b^	2.06 ^ab^	2.15 ^a^	2.16 ^a^	0.02	<0.01	0.08
n-3: n-6 ratio	0.03 ^b^	0.08 ^a^	0.091 ^a^	0.097 ^a^	0.008	<0.01	0.03

^a, b^ Means within the same row carrying different superscripts are significantly different (*p* < 0.05). ALA: alpha-linolenic acid (18:3 n − 3), EPA: eicosapentaenoic acid (20:5 n − 3), DPA: docosapentaenoic acid (22:5 n − 3), DHA: docosahexaenoic acid (22:6 n − 3), LA: linoleic acid (18:2 n − 6), AA: arachidonic acid (20:4 n − 6), n − 3 (% of total fatty acids): omega-3 PUFA, n − 6 (% of total fatty acids): omega-6 PUFA.

**Table 6:** Impact of lavender essential oil addition on the liver and kidney function of broiler chickens.

Parameters	Lavender Essential Oil, mg/kg				SEM	*p*-Value	
	0	200	400	600		Linear	Quadratic
ALT (U/L)	5.1 ^b^	7.2 ^ab^	7.34 ^ab^	7.65 ^a^	0.42	0.03	0.12
AST (U/L)	50.3	53.63	51.2	54.64	1.44	0.44	0.39
Creatinine (mg/dl)	0.70 ^ab^	0.71 ^ab^	0.69 ^b^	0.73 ^a^	0.01	0.02	0.08
Uric acid (mg/dl)	2.75	2.83	3.34	3.19	0.09	0.07	0.47

^a, b^ Means within the same row carrying different superscripts are significantly different (*p* < 0.05). Aspartate aminotransferase (AST). Alanine aminotransferase (ALT).

**Table 7:** Impact of lavender essential oil addition on broiler chickens’ serum antioxidant activity and inflammatory responses.

Parameters	Lavender Essential Oil, mg/kg				SEM	*p*-Value	
	0	200	400	600		Linear	Quadratic
TAC (U/mL)	10.21 ^c^	11.49 ^b^	13.06 ^a^	13.75 ^a^	0.43	<0.01	0.29
CAT (U/mL)	2.80 ^c^	3.87 ^bc^	5.51 ^ab^	6.22 ^a^	0.44	<0.01	0.69
SOD (U/mL)	136.31 ^c^	145.52 ^bc^	154.1 ^ab^	160.84 ^a^	2.98	<0.01	0.64
MDA (nmol/mL)	6.66 ^a^	4.12 ^b^	3.22 ^b^	3.39 ^b^	0.45	<0.01	0.01
IL1β ug/mL	142 ^c^	155 ^b^	161.33 ^ab^	170.66 ^a^	3.37	<0.01	0.54
IFN- γ (pg/mL)	7.32 ^c^	11.23 ^b^	13.48 ^ab^	14.82 ^a^	0.90	<0.01	0.90

^a, b, c^ Means within the same row carrying different superscripts are significantly different (*p* < 0.05). TAC: total antioxidant capacity, CAT: catalase, SOD: superoxide dismutase, MDA: malondialdehyde. IL1β: interleukine 1 beta. IFN-γ: interferon-γ.

## Data Availability

The datasets used and analyzed during the current study are available from the corresponding author upon reasonable request.

## References

[1] Amer S.A., Al-Khalaifah H.S., Gouda A., Osman A., Goda N.I., Mohammed H.A., Darwish M.I., Hassan A.M., Mohamed S.K.A. (2022). Potential effects of anthocyanin-rich roselle (*Hibiscus sabdariffa* L.) extract on the growth, intestinal histomorphology, blood biochemical parameters, and the immune status of broiler chickens. Antioxidants.

[2] Amer S.A., Mohamed W.A., Gharib H.S., Al-Gabri N.A., Gouda A., Elabbasy M.T., El-Rahman A., Ghada I., Omar A.E. (2021). Changes in the growth, ileal digestibility, intestinal histology, behavior, fatty acid composition of the breast muscles, and blood biochemical parameters of broiler chickens by dietary inclusion of safflower oil and vitamin C. BMC Vet. Res..

[3] Omar A.E., Al-Khalaifah H.S., Osman A., Gouda A., Shalaby S.I., Roushdy E.M., Abdo S.A., Ali S.A., Hassan A.M., Amer S.A. (2022). Modulating the Growth, Antioxidant Activity, and Immunoexpression of Proinflammatory Cytokines and Apoptotic Proteins in Broiler Chickens by Adding Dietary Spirulina platensis Phycocyanin. Antioxidants.

[4] Gou Z., Cui X., Li L., Fan Q., Lin X., Wang Y., Jiang Z., Jiang S. (2020). Effects of dietary incorporation of linseed oil with soybean isoflavone on fatty acid profiles and lipid metabolism-related gene expression in breast muscle of chickens. Animal.

[5] Kishawy A.T., Amer S.A., Abd El-Hack M.E., Saadeldin I.M., Swelum A.A. (2019). The impact of dietary linseed oil and pomegranate peel extract on broiler growth, carcass traits, serum lipid profile, and meat fatty acid, phenol, and flavonoid contents. Asian-Australas. J. Anim. Sci..

[6] WHO (2020). The State of Food Security and Nutrition in the World 2020: Transforming Food Systems for Affordable Healthy Diets.

[7] Berzaghi P., Dalle Zotte A., Jansson L., Andrighetto I. (2005). Near-infrared reflectance spectroscopy as a method to predict chemical composition of breast meat and discriminate between different n-3 feeding sources. Poult. Sci..

[8] Amer S.A., Tolba S.A., AlSadek D.M., Abdel Fattah D.M., Hassan A.M., Metwally A.E. (2021). Effect of supplemental glycerol monolaurate and oregano essential oil blend on the growth performance, intestinal morphology, and amino acid digestibility of broiler chickens. BMC Vet. Res..

[9] Adaszyńska-Skwirzyńska M., Szczerbińska D. (2017). Use of essential oils in broiler chicken production—A review. Ann. Anim. Sci..

[10] Lee J.-W., Kim D.-H., Kim Y.-B., Jeong S.-B., Oh S.-T., Cho S.-Y., Lee K.-W. (2020). Dietary encapsulated essential oils improve production performance of coccidiosis-vaccine-challenged broiler chickens. Animals.

[11] Han X., Parker T.L., Dorsett J. (2017). An essential oil blend significantly modulates immune responses and the cell cycle in human cell cultures. Cogent Biol..

[12] Amer S.A., Metwally A.E., Ahmed S.A. (2018). The influence of dietary supplementation of cinnamaldehyde and thymol on the growth performance, immunity and antioxidant status of monosex Nile tilapia fingerlings (*Oreochromis niloticus*). Egypt. J. Aquat. Res..

[13] Abbasi M.A., Ghazanfari S., Sharifi S.D., Ahmadi Gavlighi H. (2020). Influence of dietary plant fats and antioxidant supplementations on performance, apparent metabolizable energy and protein digestibility, lipid oxidation and fatty acid composition of meat in broiler chicken. Vet. Med. Sci..

[14] Zeng Z., Zhang S., Wang H., Piao X. (2015). Essential oil and aromatic plants as feed additives in non-ruminant nutrition: A review. J. Anim. Sci. Biotechnol..

[15] Abdel-Wareth A., Kehraus S., Hippenstiel F., Südekum K.-H. (2012). Effects of thyme and oregano on growth performance of broilers from 4 to 42 days of age and on microbial counts in crop, small intestine and caecum of 42-day-old broilers. Anim. Feed. Sci. Technol..

[16] Abdel-Wareth A., Lohakare J. (2014). Effect of dietary supplementation of peppermint on performance, egg quality, and serum metabolic profile of Hy-Line Brown hens during the late laying period. Anim. Feed. Sci. Technol..

[17] Abdel-Wareth A., Lohakare J. (2020). Productive performance, egg quality, nutrients digestibility, and physiological response of bovans brown hens fed various dietary inclusion levels of peppermint oil. Anim. Feed. Sci. Technol..

[18] Abdel-Wareth A.A., Lohakare J. (2021). *Moringa oleifera* leaves as eco-friendly feed additive in diets of hy-line brown hens during the late laying period. Animals.

[19] Botsoglou N., Christaki E., Florou-Paneri P., Giannenas I., Papageorgiou G., Spais A. (2004). The effect of a mixture of herbal essential oils or α-tocopheryl acetate on performance parameters and oxidation of body lipid in broilers. S. Afr. J. Anim. Sci..

[20] Botsoglou N., Florou-Paneri P., Botsoglou E., Dotas V., Giannenas I., Koidis A., Mitrakos P. (2005). The effect of feeding rosemary, oregano, saffron and α-tocopheryl acetate on hen performance and oxidative stability of eggs. S. Afr. J. Anim. Sci..

[21] Kim N.-S., Lee D.-S. (2002). Comparison of different extraction methods for the analysis of fragrances from Lavandula species by gas chromatography–mass spectrometry. J. Chromatogr. A.

[22] Lu H., Li H., Lu H., Li X., Zhou A. (2010). Chemical composition of lavender essential oil and its antioxidant activity and inhibition against rhinitis-related bacteria. Afr. J. Microbiol. Res..

[23] Białoń M., Krzyśko-Łupicka T., Nowakowska-Bogdan E., Wieczorek P.P. (2019). Chemical composition of two different lavender essential oils and their effect on facial skin microbiota. Molecules.

[24] Rabiei Z., Rafieian-Kopaei M., Mokhtari S., Shahrani M. (2014). Effect of dietary ethanolic extract of *Lavandula officinalis* on serum lipids profile in rats. Iran. J. Pharm. Res. IJPR.

[25] Jalali-Heravi M., Moazeni-Pourasil R.S., Sereshti H. (2015). Elimination of chromatographic and mass spectrometric problems in GC–MS analysis of *Lavender* essential oil by multivariate curve resolution techniques: Improving the peak purity assessment by variable size moving window-evolving factor analysis. J. Chromatogr. B.

[26] Adaszyńska-Skwirzyńska M., Szczerbińska D., Zych S. (2021). The use of lavender (*Lavandula angustifolia*) essential oil as an additive to drinking water for broiler chickens and its in vitro reaction with enrofloxacin. Animals.

[27] Giovannini D., Gismondi A., Basso A., Canuti L., Braglia R., Canini A., Mariani F., Cappelli G. (2016). *Lavandula angustifolia* Mill. essential oil exerts antibacterial and anti-inflammatory effect in macrophage mediated immune response to *Staphylococcus aureus*. Immunol. Investig..

[28] Jamróz E., Juszczak L., Kucharek M. (2018). Investigation of the physical properties, antioxidant and antimicrobial activity of ternary potato starch-furcellaran-gelatin films incorporated with lavender essential oil. Int. J. Biol. Macromol..

[29] Rashed M.M., Tong Q., Nagi A., Li J., Khan N.U., Chen L., Rotail A., Bakry A.M. (2017). Isolation of essential oil from *Lavandula angustifolia* by using ultrasonic-microwave assisted method preceded by enzymolysis treatment, and assessment of its biological activities. Ind. Crops Prod..

[30] Adaszyńska-Skwirzyńska M., Szczerbińska D. (2019). The effect of lavender (*Lavandula angustifolia*) essential oil as a drinking water supplement on the production performance, blood biochemical parameters, and ileal microflora in broiler chickens. Poult. Sci..

[31] Aviagen R. Ross Broiler Management Manual, 2009. http://pt.aviagen.com/assets/Tech_Center/Ross_Broiler/Ross_Broiler_Manual_.

[32] Brody S. (1945). Bioenergetics and growth; with special reference to the efficiency complex in domestic animals. Q. Rev. Biol..

[33] McDonald P., Edwards R., Greenhalgh J. (1973). Animal Nutrition: By P. McDonald, RA Edwards and JFD Greenhalgh.

[34] Belitz H.-D., Grosch W., Schieberle P. (2009). Meat. Food Chem.Springer.

[35] AOAC (2000). Official Methods of Analysis of AOAC International.

[36] Association A.V.M. (2013). AVMA Guidelines for the Euthanasia of Animals: 2013 Edition.

[37] Mcdonald R.E., Hultin H.O. (1987). Some characteristics of the enzymic lipid peroxidation system in the microsomal fraction of flounder skeletal muscle. J. Food Sci..

[38] Rice-Evans C., Miller N.J. (1994). [241 Total antioxidant status in plasma and body fluids. Methods Enzymol..

[39] Aebi H. (1984). [13] Catalase in vitro. Methods in Enzymology.

[40] Nishikimi M., Rao N.A., Yagi K. (1972). The occurrence of superoxide anion in the reaction of reduced phenazine methosulfate and molecular oxygen. Biochem. Biophys. Res. Commun..

[41] Suvarna S., Layton C., Bancroft J. (2013). The hematoxylins and eosin. Bancroft’s Theory and Practice of Histological Techniques.

[42] Saber S., Khalil R.M., Abdo W.S., Nassif D., El-Ahwany E. (2019). Olmesartan ameliorates chemically-induced ulcerative colitis in rats via modulating NFκB and Nrf-2/HO-1 signaling crosstalk. Toxicol. Appl. Pharmacol..

[43] Rizzardi A.E., Johnson A.T., Vogel R.I., Pambuccian S.E., Henriksen J., Skubitz A.P., Metzger G.J., Schmechel S.C. (2012). Quantitative comparison of immunohistochemical staining measured by digital image analysis versus pathologist visual scoring. Diagn. Pathol..

[44] Brenes A., Roura E. (2010). Essential oils in poultry nutrition: Main effects and modes of action. Anim. Feed. Sci. Technol..

[45] Šimunović K., Sahin O., Kovač J., Shen Z., Klančnik A., Zhang Q., Smole Možina S. (2020). (-)-α-Pinene reduces quorum sensing and Campylobacter jejuni colonization in broiler chickens. PLoS ONE.

[46] da Silva Rivas A.C., Lopes P.M., de Azevedo Barros M.M., Costa Machado D.C., Alviano C.S., Alviano D.S. (2012). Biological activities of α-pinene and β-pinene enantiomers. Molecules.

[47] Cabuk M., Alcicek A., Bozkurt M., Imre N. Antimicrobial properties of the essential oils isolated from aromatic plants and using possibility as alternative feed additives. Proceedings of the National Animal Nutrition Congress.

[48] Küçükyilmaz K., Kiyma Z., Çetinkaya M., Ateş A., Atalay H., Akdağ A., Bozkurt M., Gürsel F. (2017). Effect of lavender (*Lavandula stoechas*) essential oil on growth performance, carcass characteristics, meat quality and antioxidant status of broilers. S. Afr. J. Anim. Sci..

[49] Salajegheh A., Salarmoini M., Afsharmanesh M., Salajegheh M. (2018). Growth performance, intestinal microflora, and meat quality of broiler chickens fed lavender (*Lavandula angustifolia*) powder. J. Livest. Sci. Technol..

[50] Nasiri-Moghaddam H., Hassanabadi A., Bidar N. (2012). Effects of increasing levels of lavender essential oil (*Lavandula angustifolia*) on performance and hematological traits of broilers. Iran. J. Anim. Sci. Res..

[51] Zanetti E., De Marchi M., Dalvit C., Molette C., Rémignon H., Cassandro M. (2010). Carcase characteristics and qualitative meat traits of three Italian local chicken breeds. Br. Poult. Sci..

[52] Smet K., Raes K., Huyghebaert G., Haak L., Arnouts S., De Smet S. (2008). Lipid and protein oxidation of broiler meat as influenced by dietary natural antioxidant supplementation. Poult. Sci..

[53] Giannenas I., Bonos E., Skoufos I., Tzora A., Stylianaki I., Lazari D., Tsinas A., Christaki E., Florou-Paneri P. (2018). Effect of herbal feed additives on performance parameters, intestinal microbiota, intestinal morphology and meat lipid oxidation of broiler chickens. Br. Poult. Sci..

[54] Saleh H., Golian A., Kermanshahi H., Mirakzehi M. (2018). Antioxidant status and thigh meat quality of broiler chickens fed diet supplemented with α-tocopherolacetate, pomegranate pomace and pomegranate pomace extract. Ital. J. Anim. Sci..

[55] Dhama K., Latheef S.K., Mani S., Samad H.A., Karthik K., Tiwari R., Khan R.U., Alagawany M., Farag M.R., Alam G.M. (2015). Multiple beneficial applications and modes of action of herbs in poultry health and production-a review. Int. J. Pharmacol..

[56] Kartikasari L., Hughes R., Geier M., Makrides M., Gibson R. (2012). Dietary alpha-linolenic acid enhances omega-3 long chain polyunsaturated fatty acid levels in chicken tissues. Prostaglandins Leukot. Essent. Fat. Acids.

[57] Hristakieva P., Oblakova M., Mincheva N., Ivanova I., Lalev M., Ivanov N., Penchev I. (2021). Effect Of Dry Herbal Feed Additive On The Performance And Meat Quality of Turkeys Broilers. J. Hyg. Eng..

[58] Wisniak J. (1994). Potential uses of jojoba oil and meal—A review. Ind. Crops Prod..

[59] Gad H.A., Roberts A., Hamzi S.H., Gad H.A., Touiss I., Altyar A.E., Kensara O.A., Ashour M.L. (2021). Jojoba Oil: An updated comprehensive review on chemistry, pharmaceutical uses, and toxicity. Polymers.

[60] Zhang Y., Li X.-Y., Zhang B.-S., Ren L.-N., Lu Y.-P., Tang J.-W., Lv D., Yong L., Lin L.-T., Lin Z.-X. (2022). In vivo antiviral effect of plant essential oils against avian infectious bronchitis virus. BMC Vet. Res..

[61] Miguel M.G. (2010). Antioxidant and anti-inflammatory activities of essential oils: A short review. Molecules.

[62] Sandner G., Heckmann M., Weghuber J. (2020). Immunomodulatory activities of selected essential oils. Biomolecules.

[63] Chouhan S., Guleria S. (2020). Anti-inflammatory Activity of Medicinal Plants: Present Status and Future Perspectives. Botanical Leads for Drug Discovery.

[64] Imbabi T., Sabeq I., Osman A., Mahmoud K., Amer S.A., Hassan A.M., Kostomakhin N., Habashy W., Easa A.A. (2021). Impact of Fennel Essential Oil as an Antibiotic Alternative in Rabbit Diet on Antioxidant Enzymes Levels, Growth Performance, and Meat Quality. Antioxidants.

[65] Amer S.A., El-Araby D.A., Tartor H., Farahat M., Goda N.I., Farag M.F., Fahmy E.M., Hassan A.M., Abo El-Maati M.F., Osman A. (2022). Long-Term Feeding with Curcumin Affects the Growth, Antioxidant Capacity, Immune Status, Tissue Histoarchitecture, Immune Expression of Proinflammatory Cytokines, and Apoptosis Indicators in Nile Tilapia, Oreochromis niloticus. Antioxidants.

[66] Barbarestani S.Y., Jazi V., Mohebodini H., Ashayerizadeh A., Shabani A., Toghyani M. (2020). Effects of dietary lavender essential oil on growth performance, intestinal function, and antioxidant status of broiler chickens. Livest. Sci..

[67] Carrasco A., Martinez-Gutierrez R., Tomas V., Tudela J. (2016). Lavandula angustifolia and Lavandula latifolia essential oils from Spain: Aromatic profile and bioactivities. Planta Med..

[68] Bose S.K., Nirbhavane P., Batra M., Chhibber S., Harjai K. (2020). Nanolipoidal α-terpineol modulates quorum sensing regulated virulence and biofilm formation in Pseudomonas aeruginosa. Nanomedicine.

[69] Beier R.C., Byrd II J.A., Kubena L.F., Hume M.E., McReynolds J.L., Anderson R.C., Nisbet D.J. (2014). Evaluation of linalool, a natural antimicrobial and insecticidal essential oil from basil: Effects on poultry. Poult. Sci..

[70] Puvača N., Čabarkapa I., Petrović A., Bursić V., Prodanović R., Soleša D., Lević J. (2019). Tea tree (*Melaleuca alternifolia*) and its essential oil: Antimicrobial, antioxidant and acaricidal effects in poultry production. World’s Poult. Sci. J..

